# Medicine 4.0: New Technologies as Tools for a Society 5.0

**DOI:** 10.3390/jcm9072198

**Published:** 2020-07-12

**Authors:** Giuseppe Ioppolo, Franck Vazquez, Michael G. Hennerici, Emmanuel Andrès

**Affiliations:** 1 Department of Economics, University of study of Messina, Via dei Verdi, 75, 98122 Messina, Italy; 2 MDPI AG, St. Alban-Anlage 66, CH-4052 Basel, Switzerland; vazquez@mdpi.com; 3 Department of Neurology, Medical Faculty Mannheim, University Hospital Mannheim–University Heidelberg, D-68167 Mannheim, Germany; m.g.hennerici@eurostroke.eu; 4 Pôle M.I.R.N.E.D. et Service du Service de Médecine Interne, Diabète et Maladies Métaboliques Hôpitaux Universitaires de Strasbourg, Faculté de Médecine-Université de Strasbourg, 1 porte de l’Hôpital, 67000 Strasbourg, France; Emmanuel.ANDRES@chru-strasbourg.fr

**Keywords:** Covid-19, medicine 4.0, emerging technology, society 5.0

## Abstract

Are new technologies in the medicine sector a driver to support the development of a society 5.0? Innovation pushes the artisan to become smart and lean, customer-oriented but within a standardized environment of production, maintaining and ensuring the quality of the product. An artisan is a user and innovator, as an essential part of the industrial chain. In the healthcare sector, the doctor is the industrial artisan, and medicine can be considered as an example of a smart tool, strongly tailored, that embeds the innovation of materials, nano-devices, and smart technology (e.g., sensors and controllers). But how much of society is ready to host smart technology “on board”, becoming “on life”, constantly connected with remote controls that allow us to monitor, gather data, and, in any case, act, with preventive healthcare solutions? After a short overview of the medicine sector, a preliminary, tentative link between technological innovation and the healthcare sector allows us to adopt several outlooks on how to change research, always more transdisciplinary, combining science with social science in order to remain human-centered.

The lockdown due to the Covid-19 (Coronavirus) pandemic changed society’s behaviors and introduced new risks in the medical sector. In the new contactless scenario, healthcare facilities have been affected by proximity risks. Simultaneously with this, the continuous process of innovation in advanced technologies, thanks to the forced introduction of artificial intelligence, not only in production models but also in the relationship between product, function and use, has had an ever-greater impact on both the new model of industry 4.0 and society 5.0 [1]. One of these application fields is the healthcare sector. Covid-19 risks and the management of new healthcare processes allowed us to rethink the healthcare model.

In order to understand the main innovation cycles, briefly, four representative eras can be seen in the medicine world. While medicine 1.0 has been largely defined by the treatment of consolidated illnesses, in medicine 2.0, patient-centered human healthcare begins to be technology-based, result-oriented, and focused on prevention, with significant benefits for the person, the supplier and the company.

Then, medicine 3.0 represented the moment of transition and overcoming barriers connected to the transformation of the way of operating, through the forced introduction of technology in the working process. The lean models, in terms of both production and management, push towards total flexibility, which, through the innovative use of, for example, “smart materials”, transforms the healthcare sector, introducing a digital model.

Emerging technologies certainly represent a central pillar of medicine 4.0 [2]. During this pandemic age, the smart supply chain—for instance, in the procurement and management of medicines; the introduction of robotics to support Covid-19 hospitals/departments, from triage to several activities such as checking and therapeutic administration during patient management, but also 3D printers used for healthcare protection or for component supplies; a digital environment to support information exchange between the patient and the doctor, but also within the different departments intra- and inter-hospitals, and finally between patient and family; sensors and smart technologies are all key factors/tools in this transition through a medicine 4.0 [3]. Social distancing is the new normal.

Indeed, in manufacturing, also due to digitalization, it is now possible to create virtual copies of the patient, on the basis of which we proceed to the creation of models through so-called “digital design” processes, reducing the possibility of error to zero while reducing costs and improving the quality of performance and yield for patients [4]. The relationships between robotic tools and sensors for scanning, digitalization, and data analysis, design and production using CAD/CAM, and finally 3D printing, are processed with a complete reduction in human interaction [5,6]. New immaterial and digital factories, based on smart working and networking, are reengineering the production system.

This revolution, therefore, gives life to what is increasingly identified as the evolution of the health profession, now based on innovative and promising technological resources that culminate in healthcare’s solid professional skills. This translates into an improved and safer service. Consequently, how can medicine 4.0 adapt and advance in the rapidly changing digital world?

In the new scenarios of medicine 4.0, the role of Artificial Intelligence (AI) will be the center of gravity, which responds to the need for flexibility and at the same time quality of service, and is increasingly customer oriented, in a one-to-one mode, as in the AI-based medical imaging analysis which has been introduced in several Covid-19 centers [7]. The ability to gather and manage large amounts of data (the huge dimension of healthcare information on the patient), processed from a descriptive approach to a predictive one, introduces an emerging role to all the smart technologies as machine learning and AI [8].

As the basis of these new scenarios, holistic relationships that increase the value chain must be considered; that is, the increasing computing power and memory storage of devices (nano devices); an extensive concentration on the Internet of Things (IoT), which collects data from connected devices; the ability to implement advanced algorithms also with predictive capabilities; the possibility of upgrading the AI functionality even to existing products (application processing, interface operability), etc. [9].

Technology can contribute to different hospital processes—for example, robotics and AGV (automated guided vehicle) and autonomous systems can contribute to waste recovery and management, maintaining a safe supply chain and reducing contact risks and Covid-19 exposure.

From hospital to home health care, using a combination of mHealth devices (mobile devices), data science tools and behavioral strategies, the patient takes an active role in data collection. Automatic systems for monitoring and collecting information will contribute to overcoming the current limitations of AI, i.e., the insufficiency and current accuracy of the data. Combining intelligent implantable systems, due to the continuous miniaturization of electronics, also within a biological environment (body sensors and dedicated AI chips), with additive technology, it is possible to produce super-tailored smart tools. Therefore, biomedical and health systems provide new drivers for the medicine 4.0 transition. Thanks to dedicated sensors, an intelligent agent can assess a patient’s clinical condition, based on data mining processes, and select autonomously or according to the specialist indications the best decision (i.e., pharmacological therapy) and intervene through actuators.

However, the trends described above clearly indicate the inevitable transformation of the healthcare model, which must rest on an evolved social model, into a society 5.0.

Society 5.0 is the last generation (also called Y Generation), younger than millennials and totally digital native. In order to realize meaningful human–machine interfaces (HMIs), an “ambient intelligent system” must be performed, where people can even go so far as to give up control in the decision-making process, in an environment which has full confidence in the extended use of technology.

This social model will need to have full access to the reach and capabilities of digital technologies in order to remain “on life”, the new trend to be always connected. Consequently, this will be a social model that relies on machine learning systems, which, through machine learning, can manage a patient and the relationship with his family doctor without human physical interaction, autonomously and automatically.

Technology and mass data are therefore profoundly reshaping society. As the huge quantities of digital data generated by mobile technologies begin to merge with the clinical, molecular, and contextual data of thousands to millions of patients, the healthcare sector will face the challenges and opportunities presented by the volume, variety, and complexity of big data.

A digital world needs a digital community, and this is also true in the healthcare sector; the big data paradigm could be a strategic resource but it will have an impact on patients, clinical systems, and healthcare management [10].

Beside smart hospitals, medicine 4.0 combines all the current and future emerging technologies, from the detection and diagnostic phases to the monitoring and treatment phases of the patient. This appears fruitful for the protection of both patients and healthcare professionals, building a safer environment, especially for Covid-19 patients in whom the infection risk is very high [3].

Smart technologies can be configured as a precursor to the identification of diseases, also facilitating the systematic coordination of health services, creating a model of personalized, more efficient “health” that is capable of managing the complexity of information and related therapies, thus reducing the costs. Together with the oral microbiome, metabolomic, genetic, and immune biomarkers, these datasets provide a very rich data resource at the molecular, genetic, cellular, organ, and system levels that requires a systematic application of data science. The next technological challenge is to extract the information contained and transform the raw data into feasible clinical knowledge and insights.

In the near future, to improve oral health, reducing unnecessary costs, both at the individual patient and the health system levels, will play a central role in the integration of digital technologies, which will allow the development of personalized strategies, introducing the remote monitoring of patients and the acquisition, analysis, and return of big data [11].

In our future outlooks, medicine 4.0 must be a proactive model, capable of responding to pressure, and at the same time it must take advantage of the strategic opportunities contained in order to create added value for the patient, the supplier, and the company. The five Ps of Medicine— the predictive, preventive, personalized, participatory, and purpose-driven approach—represent a strategic and innovative challenge, and adopting e-health tools and solutions guarantees a sustainable transition towards a new techno-digital, patient-centered environment [12]. This is the emerging issue that must be addressed in order to realize a meaningful integrative medical approach.

The future is now, but first, new research has to accomplish the following: (1) expand on dynamic processes and industry outcomes; (2) offer new reflections on artificial intelligence applied in medicine 4.0; (3) provide a comparative analysis of dynamics across social, technological, and institutional settings; (4) examine the barriers to the comparative analysis of emerging technologies (big data, machine learning, IoT, etc.) applied to medicine 4.0; (5) present other conceptual configurations in medicine 4.0. Secondly, research on the institutions, public and private, as a new model of governance, has to support the implementation of digital and smart solutions only by supporting quality of life, overcoming prejudices and social barriers.
